# The Effects of Li/Nb Ratio on the Preparation and Photocatalytic Performance of Li-Nb-O Compounds

**DOI:** 10.1186/s11671-017-2273-8

**Published:** 2017-08-15

**Authors:** Haifa Zhai, Hairui Liu, Hongjing Li, Liuyang Zheng, Chunjie Hu, Zhao Wang, Jingjing Qi, Jien Yang

**Affiliations:** 1 Henan Key Laboratory of Photovoltaic Materials, College of Physics and Materials Science, Henan Normal University, Xinxiang, 453007 People’s Republic of China; 2 National Laboratory of Solid State Microstructures, Nanjing University, Nanjing, 210093 People’s Republic of China

**Keywords:** Lithium Niobate, Hydrothermal, Porous Materials, Photocatalysis

## Abstract

The effects of Li/Nb ratio on the preparation of Li-Nb-O compounds by a hydrothermal method were studied deeply. Li/Nb ratio has a great impact on the formation of LiNbO_3_; the ratio smaller than 3:1 is beneficial to the formation of LiNbO_3_, while larger than 3:1, forms no LiNbO_3_ at all and the morphology and chemical bond of Nb_2_O_5_ raw material are totally modified by Li ions. The reason can be attributed to the large content of LiOH, which is beneficial to form Li_3_NbO_4_ not LiNbO_3_, and also, even if LiNbO_3_ particle locally forms, it is easily dissolved in LiOH solution with strong alkalinity. Pure LiNb_3_O_8_ powders are obtained with two absolutely opposite Li/Nb ratios: 8:1 and 1:3; the former shows a unique porous and hollow structure, quite different from the particle aggregation (the latter shows). Compared with Li/Nb = 1:3, the 4.2 times higher photocatalytic performance of LiNb_3_O_8_ (Li/Nb = 8:1) are observed and it can be attributed to the unique porous and hollow structure, which provides a high density of active sites for the degradation of MB. Compared to LiNbO_3_, the improved photocatalytic performance of LiNb_3_O_8_ can be attributed to its layered structure type with the reduced symmetry enhancing the separation of electrons and holes.

## Background

Niobium compounds, a very versatile group of materials, including niobium oxides, alkali niobates, and columbite niobates, exhibit many interesting physical properties and have been widely studied in many fields, such as catalysis [1–3], memristors [4], dye-sensitized solar cells [5], optical devices, and others [6, 7]. LiNbO_3_, as one of the most famous alkali niobates, presents prominent properties such as electro-optical and nonlinear optical behaviors, pyroelectricity, and piezoelectricity, and it is mainly used as optical modulators, waveguides, acoustic wave transducers, et al. in optical devices.

For environmental remediation and clean energy applications, niobates, such as (Na, K)NbO_3_ [8], BiNbO_4_ [9], LiNbO_3_ [10], and LiNb_3_O_8_ [11], have been deeply investigated, owing to their special distorted [NbO_6_] octahedral structures which favor a possible delocalization of charge carriers [12]. Secondly, the conduction bands consisting of Nb4d orbitals located at a more negative state of redox potential of H+/H_2_ promote the separation and transfer of photo-induced charge carriers and result in high photocatalytic activities [13]. Among these materials, LiNb_3_O_8_ displays unique performances. As a novel lithium-ion battery (LIB) anode material, the theoretical capacity of LiNb_3_O_8_ is 389 mAh/g assuming two-electron transfers (Nb^5+^ → Nb^3+^), larger than many other anode materials, such as Li_4_Ti_5_O_12_ [14, 15]. Used for supercapacitor devices, LiNb_3_O_8_ nanoflakes show excellent cycle stability with negligible specific capacitance decrease even after 15,000 cycles [16]. Also, it is used as an efficient photocatalyst in the applications of hydrogen generation and degradation of organic pollutants. Pure LiNb_3_O_8_ is a highly active UV-photocatalyst for water reduction producing 83.87 μmol of hydrogen in 1 h, and it does not produce hydrogen under visible-light irradiation due to its large band gap (i.e., 3.9 eV) and inability to absorb visible light [17, 18]. LiNb_3_O_8_ nanoflakes show fast decolorization of toluidine blue O (TBO) dye under UV light compared to commercial TiO_2_ powders [13].

At most time, the appearance of LiNb_3_O_8_ is recognized as an impurity phase during the preparation of LiNbO_3_, especially in film samples, owing to high annealing temperature or inhomogeneous distribution of Li element in precursors [19, 20]. Due to the difficulty to prepare a pure phase, LiNb_3_O_8_ has been rarely studied, while for LiNbO_3_ powders, the preparation technologies are various, such as sol-gel [19], hydrothermal [21], and laser irradiation methods [22]. Hydrothermal method is widely used to synthesize nanomaterials with advantages such as low temperature, environmental friendliness, and homogenous particle-size distribution, which can efficiently avoid the variation of Li/Nb molar ratio without going through high temperatures. As for hydrothermal method, the parameters of reaction temperature, raw material ratio, and holding time play important roles in determining the as-obtained materials, while the research of Li/Nb ratio much larger than 1:1 in the preparation of Li-Nb-O compounds has not been reported before.

In this paper, the effects of Li/Nb ratio on the preparation of Li-Nb-O compounds by a hydrothermal method were studied deeply. A series of analytical techniques were used to characterize the crystallinity, morphology, and chemical composition of the Li-Nb-O samples, especially the changes before and after the hydrothermal reaction. Pure LiNb_3_O_8_ and LiNbO_3_ photocatalysts were prepared, and the photocatalytic performance was studied with the effect of Li/Nb ratio in raw materials.

## Methods

The preparation of Li-Nb-O compounds was carried out by the hydrothermal method using lithium hydroxide monohydrate (LiOH·H_2_O; Aladdin, ACS, ≥ 98.0%) and niobium pentaoxide (Nb_2_O_5_; Aladdin, AR, 99.9%) as starting materials. Firstly, 3.5 mmol of Nb_2_O_5_ was dispersed into 35 ml deionized water with a certain amount of LiOH·H_2_O under magnetic stirring. The mole ratios of Li:Nb are 1:3, 1:1, 2:1, 3:1, 4:1, 5:1, 6:1, 7:1, and 8:1; as the results of the samples prepared with ratios of 4:1, 5:1, 6:1, and 7:1 are similar, only the ratios of Li:Nb = 4:1 and 7:1 are shown below. The suspension solutions were put into 50-mL Teflon-lined hydrothermal synthesis autoclave reactors and maintained at 260 °C for 24 h, then cooled down naturally to room temperature. The as-obtained powders were then washed with deionized water and ethanol for several times and dried at 60 °C. Finally, the products were calcined at various temperatures from 500 to 800 °C for 2 h with a ramp rate of 5 °C/min.

The X-ray diffraction (XRD) patterns were recorded using a Bruker D8 Discover diffractometer with Cu *Kα* radiation (40 kV, 40 mA). The morphologies of the samples were characterized by field emission scanning electron microscope (FESEM; JSM-6700F). Chemical bonds were analyzed by Fourier-transformed infrared spectroscopy (FTIR) in the range of 2000–650 cm^−1^. X-ray photoelectron spectroscopy (XPS) analysis was performed on a Thermo-Fisher Escalab 250Xi instrument to characterize the chemical component of Li-Nb-O compounds. The specific surface area was measured on a surface area apparatus (Micromeritics ASAP 2460) at 77 K by N_2_ adsorption/desorption method (BET method). The photoluminescence (PL) spectra were detected using an F-280 fluorescence spectrophotometer with an excitation wavelength of 320 nm.

To evaluate the photocatalytic performance of Li-Nb-O compounds, the degradation of methylene blue (MB) aqueous solution (5 mg/L) was carried out under irradiation of a 500 W Hg lamp at a natural pH value. Fifty milligrams of powders were dispersed into 50 mL of MB aqueous solution. Before the irradiation, the suspension was stirred in dark for 1 h to achieve adsorption equilibrium. Then, the suspension was irradiated by the Hg lamp. The concentration of residual MB was analyzed with an interval of 30 min using an ultraviolet-visible near-infrared (UV-vis-NIR) spectrophotometer at 665 nm.

## Results and Discussion

**Fig. 1:**
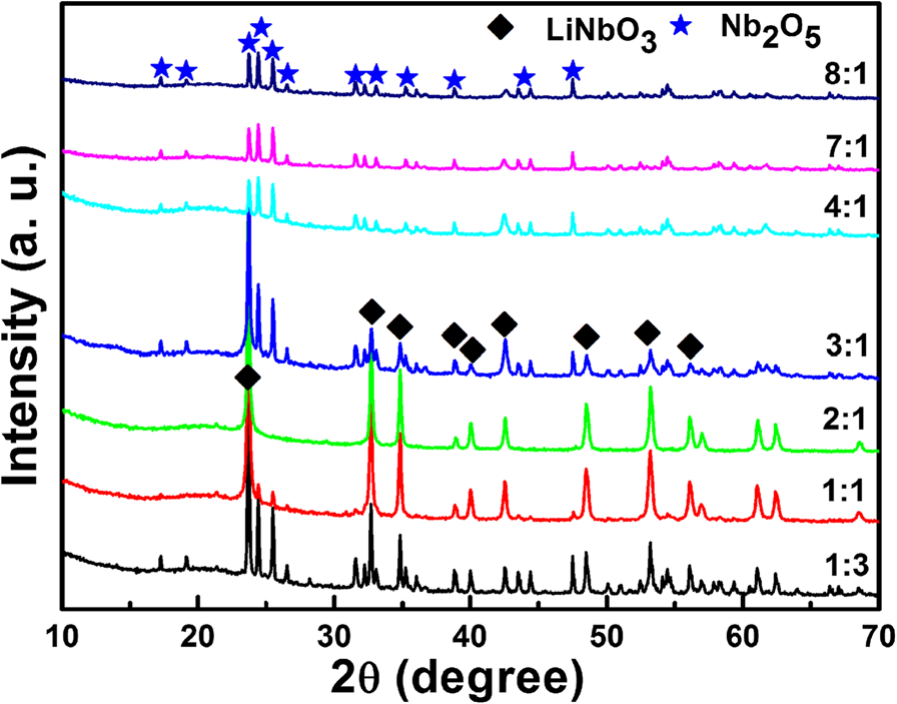
XRD patterns of the Li-Nb-O powders obtained after hydrothermal reaction with different Li/Nb mole ratios

The XRD patterns of the products obtained after hydrothermal reaction with different Li/Nb mole ratios are shown in Fig. 1. It is obvious that pure LiNbO_3_ phase (JCPDF, No. 20-0631) is obtained with Li:Nb = 2:1. For the ratio of Li/Nb smaller than 2:1, such as 1:1 or 1:3, the main phase is still LiNbO_3_, accompanied with the residual of Nb_2_O_5_ (JCPDF, No. 37-1468), which means that the Li content is not sufficient to fully react with Nb_2_O_5_ to form LiNbO_3_. When we increase the Li content largely, an amazing phenomenon occurs: there is no LiNbO_3_ formed at all after the hydrothermal reaction, as clearly shown in Fig. 1. When the ratio of Li/Nb is 4:1 or larger, only Nb_2_O_5_ phase exists in XRD patterns, no other impurities detected. Is the Li ion washed away during the washing process? Just like the former literature reported [23].

**Fig. 2:**
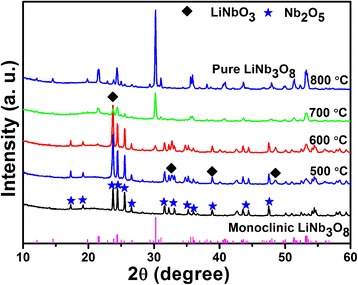
XRD patterns of the Li-Nb-O powders (mole ratio of Li:Nb = 8:1) calcined at different temperatures for 2 h

To illustrate the phase evolution when the Li/Nb ratio is large enough, the products obtained by the hydrothermal method, using Li/Nb = 8:1 as an example, are calcined at different temperatures and the XRD patterns are shown in Fig. 2. When the products are calcined at 500 and 600 °C, a new phase LiNbO_3_ appears which proves that a Li element truly exists in the products obtained just after the hydrothermal reaction, though not detected by XRD. Also, a diffraction peak at 30.26° appears at 600 °C, which can be indexed as (410) plane of monoclinic LiNb_3_O_8_. The reaction can be described by the following Eq. (1) [24]:

1 $$ {\mathrm{LiNb}\mathrm{O}}_3+{\mathrm{Nb}}_2{\mathrm{O}}_5\to {\mathrm{LiNb}}_3{\mathrm{O}}_8 $$

At 700 °C, the monoclinic LiNb_3_O_8_ is the predominant phase with almost negligible impurity. The pure phase of LiNb_3_O_8_ is obtained at 800 °C with all the diffraction peaks indexed to the monoclinic phase (JCPDF, No. 36-0307), a space group of P21/a, which provides a new way to prepare LiNb_3_O_8_ compounds.

**Fig. 3:**
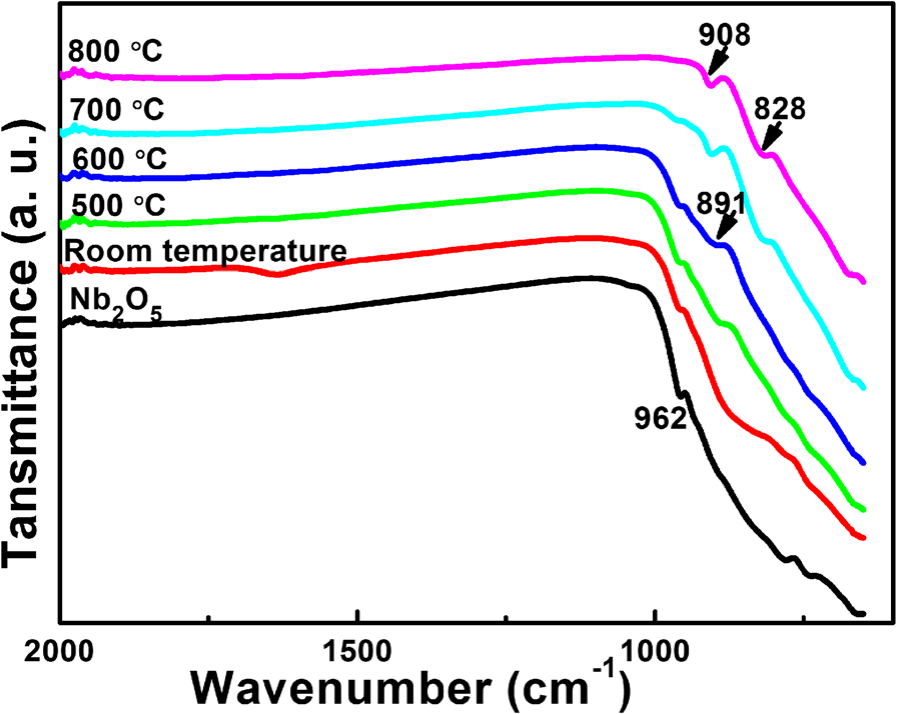
FTIR spectra of Nb_2_O_5_ raw materials and Li-Nb-O powders (mole ratio of Li:Nb = 8:1) calcined at different temperatures

FTIR test is also performed to study the phase evolution of the products with Li:Nb = 8:1, as shown in Fig. 3. The raw material Nb_2_O_5_ is tested as a reference. In Fig. 3, the band at 962 cm^−1^ due to the stretching vibrations of Nb = O in Nb_2_O_5_ is existent until 700 °C [25]. After hydrothermal reaction, no other bands detected at this range means the only niobate is still Nb_2_O_5_. When the calcination temperature is 500 and 600 °C, a new band at 891 cm^−1^ appears, while disappears at 700 °C, consistent with the XRD results of the formation and reaction of LiNbO_3_. At 700 and 800 °C, the bands at 908 and 828 cm^−1^ correspond to the formation of LiNb_3_O_8_ compounds [26, 27]. The FTIR results are well consistent with the XRD results of Fig. 2.

Based on the results, we can conclude that Li/Nb ratio has a great impact on the formation of LiNbO_3_; the ratio smaller than 3:1 is beneficial to the formation of LiNbO_3_, while larger than 3:1, no LiNbO_3_ forms at all. Based on the diagram, the congruent Li content is 97.2 mol% of the Nb content for the preparation of perfect single-phase LiNbO_3_, and the excess or deficiency of the Li content is compensated by the formation of Li_3_NbO_4_ or LiNb_3_O_8_ phase [28]. The large excess of LiOH is beneficial to form Li_3_NbO_4_ not LiNbO_3_, while no Li_3_NbO_4_ phase is observed after hydrothermal reaction due to the insufficient reaction condition; even if the LiNbO_3_ particle locally formed, it is easily dissolved in LiOH solution with strong alkalinity [29].

**Fig. 4:**
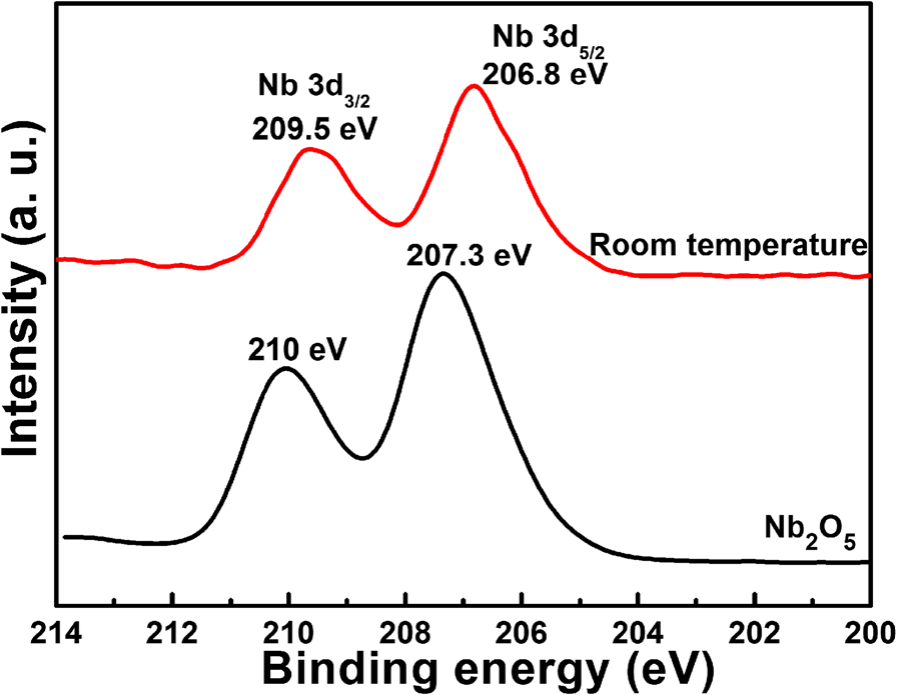
XPS spectra of Nb_2_O_5_ raw materials and the products (mole ratio of Li:Nb = 8:1) obtained after hydrothermal method

As discussed above, the Li element is not detected after the hydrothermal reaction without further calcination, while it truly exists in the products with Li:Nb = 8:1. For Nb_2_O_5_, is it still the same as the raw material after the hydrothermal reaction? The XPS test is carried out to characterize the chemical component of Nb_2_O_5_ raw material and the products obtained after hydrothermal reaction, as shown in Fig. 4. The difference of Nb 3d_3/2_ and 3d_5/2_ is the 2.7 eV for both samples, indicating the Nb^5+^ state in both samples without other reduced Nb oxides species [3]. The binding energies of Nb 3d shift towards the low binding-energy state after the hydrothermal reaction, about 0.5 eV difference. It means that the chemical environment of Nb changes, while no other compounds are formed. The change may be due to the existence of Li ions in the product; though no obvious Li-Nb-O compound is formed, the existence of Li ions with larger iconicity has strong attraction of O ions around Nb, resulting in the chemical shift of Nb 3d binding energy.

**Fig. 5:**
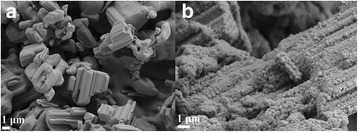
SEM images of **a** Nb_2_O_5_ raw materials and **b** the products (mole ratio of Li:Nb = 8:1) obtained after hydrothermal method

The influence of Li ions on Nb_2_O_5_ is also observed in SEM images, as shown in Fig. 5. Figure 5a is the image of Nb_2_O_5_ raw material, with irregular shape, dense structure, and length of several micrometers. After the hydrothermal reaction, the large crystal particle is divided into small particles with the maximum size of about 200 nm, though the small particles still aggregate together. From the XRD and XPS results, we know that the small particles are still Nb_2_O_5_. The change of the morphology of Nb_2_O_5_ can be attributed to the hydrothermal condition and large content of LiOH·H_2_O in raw materials.

**Fig. 6:**
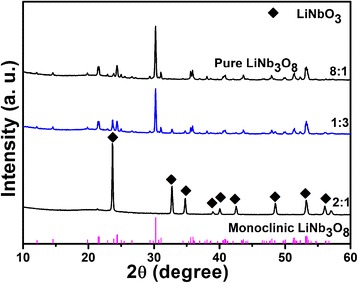
XRD patterns of three typical Li/Nb ratios products calcined at 800 °C for 2 h

The products obtained after hydrothermal reaction are calcined at 800 °C with different Li/Nb ratios. Hereafter, we choose three typical Li/Nb ratios as examples: 1:3, 2:1, and 8:1. The XRD patterns of the three samples are shown in Fig. 6. From the XRD results, pure LiNbO_3_ are prepared with Li/Nb = 2:1 and has shown no change even when calcined at 800 °C. For the preparation of another Li-Nb-O compound LiNb_3_O_8_, it can be obtained with two absolutely opposite Li/Nb ratios: 8:1 and 1:3 (designated as LiNb_3_O_8_-8:1 and LiNb_3_O_8_-1:3). For other Li/Nb ratios not shown in Fig. 6, the products calcined at 800 °C result in the formation of two mixed phases: LiNb_3_O_8_ and LiNbO_3_. Based on the XRD results, pure LiNb_3_O_8_ powders are prepared with two different Li/Nb ratios, while is there any differences between the two products?

**Fig. 7:**
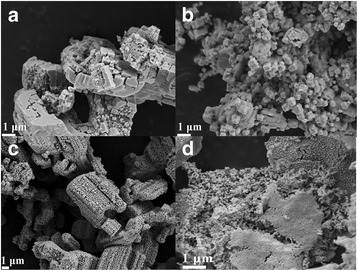
SEM images of three typical Li/Nb ratios products calcined at different temperatures: **a** 2:1 at 500 °C, **b** 1:3, **c** 8:1, and **d** 2:1 at 800 °C

The SEM images of the two products are displayed as Fig. 7b, c, respectively. As shown in Fig. 7, the morphology of LiNb_3_O_8_-1:3 are quite different with that of LiNb_3_O_8_-8:1. LiNb_3_O_8_-8:1 has a porous and hollow structure formed by LiNb_3_O_8_ nanoparticles with the length of several micrometers, similar as that of a honeycomb. It is quite different with the particle aggregation of solid-state reaction, as LiNb_3_O_8_-1:3 shown. The BET areas of LiNb_3_O_8_-8:1 and LiNb_3_O_8_-1:3 are 4.46 and 0.96 m^2^/g, respectively, the larger surface area of the former results from the porous and hollow structure. The morphology difference can be attributed to the different morphologies of the reactants: for LiNb_3_O_8_-8:1, the reactant of LiNbO_3_ is formed based on the calcinations of the products after hydrothermal reaction, the morphology of the products is shown in Fig. 5b, while for LiNb_3_O_8_-1:3, the morphology of LiNbO_3_ obtained directly after the hydrothermal reaction is hexahedron-like, as shown in Fig. 7a [21]. The formation of the porous and hollow structure for LiNb_3_O_8_-8:1 can be attributed to the lithium volatilization during the calcinations process, which is beneficial to the formation of new LiNb_3_O_8_ particles and networks between the particles [11]. For LiNbO_3_ calcined at 800 °C (i.e., Li/Nb = 2:1), its grain size is about 200 nm and the shape seems irregular, as shown in Fig. 7d; the BET area is about 3.91 m^2^/g.

**Fig. 8:**
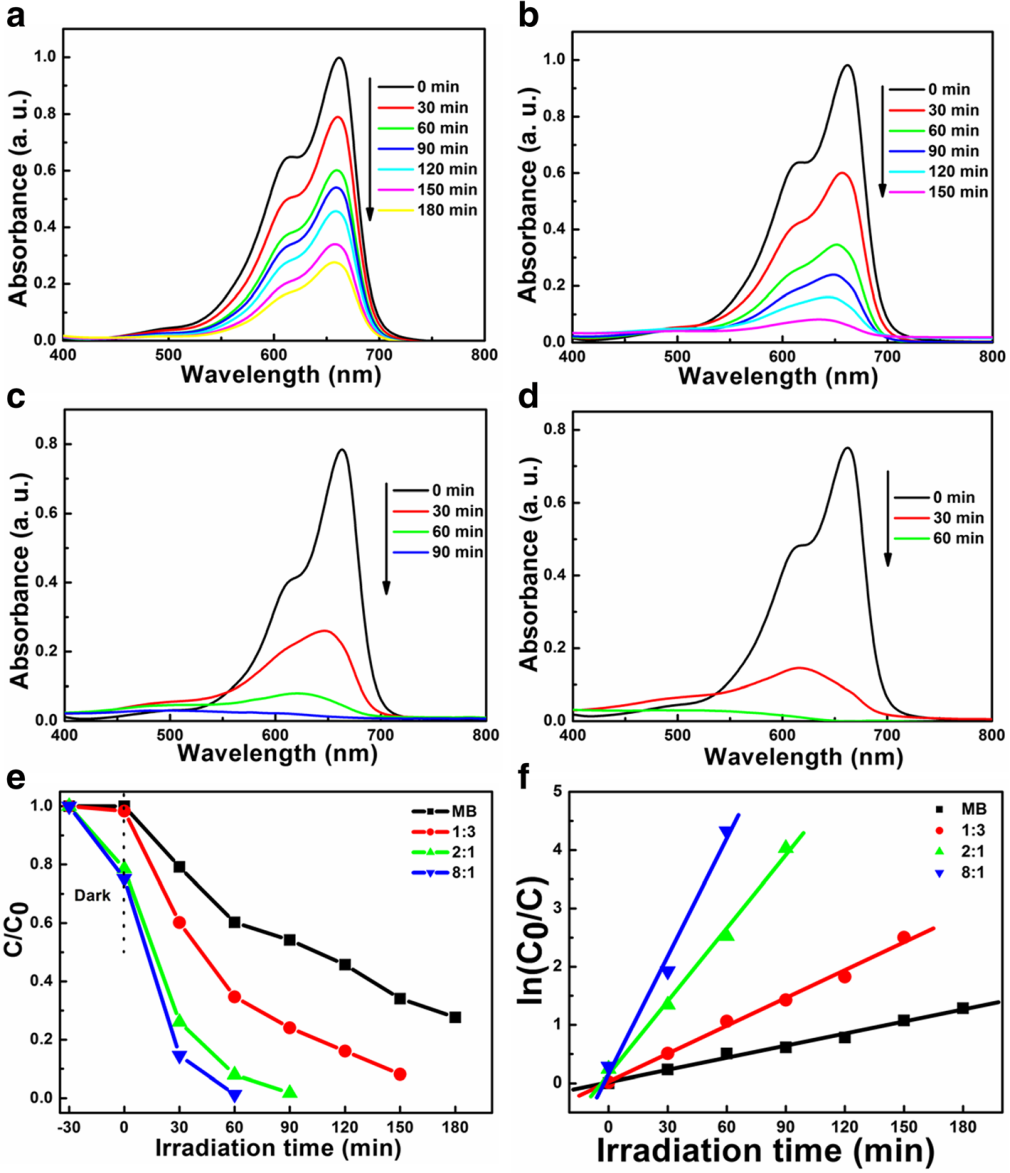
UV-vis absorption spectra of the degradation of MB: **a** without catalyst and catalyzed by **b** LiNb_3_O_8_-1:3, **c** LiNbO_3_, and **d** LiNb_3_O_8_-8:1, respectively. **e** Photo-degradation of MB and **f** kinetic fit with respect to the irradiation time using Li-Nb-O powders

The photocatalytic performances of LiNb_3_O_8_ and LiNbO_3_ are shown in Fig. 8. From the UV-vis absorption spectra of MB at the varied irradiation time (Fig. 8a–d), it is observed that the maximum absorption band (665 nm) becomes weak with the increase of the irradiation time. The degradation rate of MB is largely improved with the catalysts of LiNb_3_O_8_ and LiNbO_3_ under UV light, especially for LiNb_3_O_8_-8:1, about 85% of MB degraded after 30 min irradiation, as shown in Fig. 8e. As the photo-degradation of MB using Li-Nb-O catalysts obeys the pseudo-first-order kinetics, described by the modified Langmuir-Hinshelwood kinetics mode [30], the constants of the pseudo-first-order rate (*k*) are calculated, displayed in Fig. 8f. The obtained first-order rate constants of MB without catalysts, LiNb_3_O_8_-1:3, LiNbO_3_, and LiNb_3_O_8_-8:1 are 0.71 × 10^−2^, 1.61 × 10^−2^, 4.18 × 10^−2^, and 6.73 × 10^−2^ min^−1^, respectively. The higher the first-order rate constant is, the more outstanding the photocatalytic performance is. The *k* of LiNb_3_O_8_-8:1 is 9.5 times of MB without catalysts, 4.2 times of LiNb_3_O_8_-1:3, and 1.6 times of LiNbO_3_. Compared with LiNb_3_O_8_-1:3, the higher photocatalytic performance of LiNb_3_O_8_-8:1 can be attributed to the unique porous and hollow structure, which provides a high density of active sites for the degradation of MB [31].

**Fig. 9:**
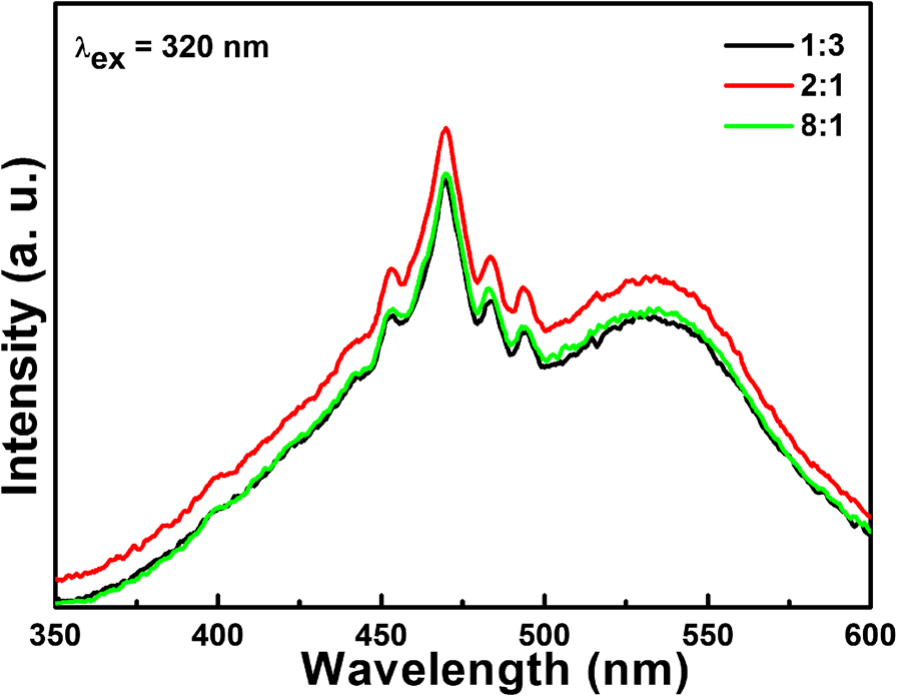
Room temperature PL spectra of LiNb_3_O_8_-1:3, LiNbO_3_, and LiNb_3_O_8_-8:1 catalysts

Compared to LiNbO_3_, the improved photocatalytic performance of LiNb_3_O_8_-8:1, which has almost the same absorption ability of MB as that of LiNbO_3_, can be attributed to its layered structure type with the reduce symmetry. The layered structure can enhance the separation of electrons and holes [32], consistent with the PL spectra, as shown in Fig. 9. At the same time, the LiNb_3_O_8_ framework is constructed by three different niobate octahedrons and Li atoms share partial octahedral sites; the higher niobate octahedral site is expected to provide more active sites for photocatalysis. Finally, the smaller energy band gap of LiNb_3_O_8_ (about 3.9 eV) than that of LiNbO_3_ (4.14 eV) means that it can utilize more incident light to participate in the photocatalytic process [33].

The separation efficiency of photogenerated carries of Li-Nb-O catalyst are investigated by PL spectra, as shown in Fig. 9. As we know, PL emission spectra mainly result from the recombination of free carriers. As seen in Fig. 9, LiNb_3_O_8_ shows smaller emitting peaks around 470 nm than LiNbO_3_. It means that LiNb_3_O_8_ has longer charge carrier lifetime and improved efficiency of interfacial charge transfer, which can be attributed to its layered structure with the reduced symmetry enhancing the separation of electrons and holes.

## Conclusions

From the results above, we can conclude that Li/Nb ratio has a great impact on the formation of LiNbO_3_; the ratio smaller than 3:1 is beneficial to the formation of LiNbO_3_, while larger than 3:1, forms no LiNbO_3_ at all and the morphology and chemical bond of Nb_2_O_5_ raw material are totally modified by Li ions. The reason can be attributed to the large content of LiOH, which is beneficial to form Li_3_NbO_4_ not LiNbO_3_, and also, even if the LiNbO_3_ particle locally forms, it is easily dissolved in LiOH solution with strong alkalinity. Pure LiNb_3_O_8_ powders are obtained with two absolutely opposite Li/Nb ratios: 8:1 and 1:3; the former shows a unique porous and hollow structure, quite different with the particle aggregation (the latter shows). Compared with Li/Nb = 1:3, higher photocatalytic performance of LiNb_3_O_8_ (Li/Nb = 8:1) are observed and it can be attributed to the unique porous and hollow structure, which provides a high density of active sites for the degradation of MB. Compared to LiNbO_3_, the improved photocatalytic performance of LiNb_3_O_8_ can be attributed to its layered structure type with the reduced symmetry enhancing the separation of electrons and holes.
